# Improved performance of a CoTe//AC asymmetric supercapacitor using a redox additive aqueous electrolyte

**DOI:** 10.1039/c7ra12919j

**Published:** 2018-02-20

**Authors:** Beirong Ye, Chao Gong, Miaoliang Huang, Yongguang Tu, Xuanqing Zheng, Leqing Fan, Jianming Lin, Jihuai Wu

**Affiliations:** Engineering Research Center of Environment-Friendly Functional Materials, Ministry of Education, Institute of Materials Physical Chemistry, College of Materials Science and Engineering, Huaqiao University Xiamen Fujian 361021 P. R. China huangml@hqu.edu.cn +86-592-6162255

## Abstract

Cobalt telluride (CoTe) nanosheets as supercapacitor electrode materials are grown on carbon fiber paper (CFP) by a facile hydrothermal process. The CoTe electrode exhibits significant pseudo-capacitive properties with a highest *C*_m_ of 622.8 F g^−1^ at 1 A g^−1^ and remarkable cycle stability. A new asymmetric supercapacitor (ASC) is assembled based on CoTe (positive electrode) and activated carbon (negative electrode), which can expand the operating voltage to as high as 1.6 V, and has a specific capacitance of 67.3 F g^−1^ with an energy density of 23.5 W h kg^−1^ at 1 A g^−1^. The performance of the ASC can be improved by introducing redox additive K_4_Fe(CN)_6_ into alkaline electrolyte (KOH). The results indicate that the ASC with K_4_Fe(CN)_6_ exhibits an ultrahigh specific capacitance of 192.1 F g^−1^ and an energy density of 67.0 W h kg^−1^, which is nearly a threefold increase over the ASC with pristine electrolyte.

## Abstract 2

The performance of an ASC with CoTe as the positive electrode can be enhanced by introducing redox additive K_4_Fe(CN)_6_ into the alkaline electrolyte.

## Graphical abstract

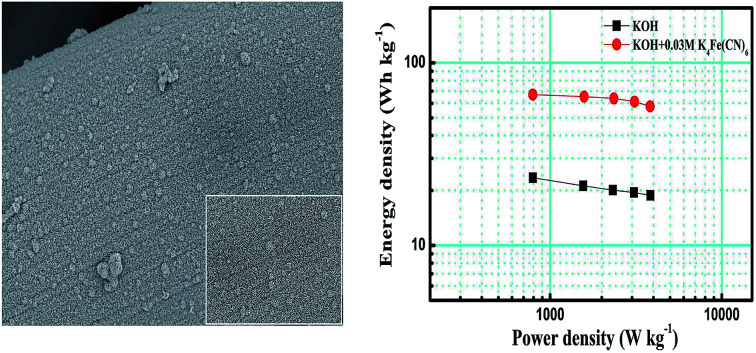

## 1. Introduction

Recently, as a new type of energy storage device, supercapacitors have received extensive attention, owing to their advantages of high specific capacitance, short charging time and excellent cycling ability.^1–3^ The energy storage mechanism of a supercapacitor is either the accumulation and electrostatic adsorption of charge on the interface between the electrode and the electrolyte (electrical double layer capacitors) or fast, reversible redox reaction on or near the surface of the electrode (pseudocapacitors).^4^ Among them, owing to their better cycle stability and higher power density than conventional batteries, electrical double layer capacitors have becoming the most common supercapacitors. However, they still present a lower energy density (3–6 W h kg^−1^).^5^ The reason is that EDLCs cannot make full use of the body phase of the electrode material for energy storage, and they are unable to expand the voltage window of the capacitor by using the difference of the positive and negative electrode. Hence, for the sake of the needs of practical application, much more efforts have been focused on improving the energy density of supercapacitors. According to the calculation formula of energy density: *E* = 1/2 *CV*^2^, the energy density of supercapacitors can be improved by increasing capacitance (*C*) or enlarging the operating voltage (*V*).^6,7^ A promising approach is to assemble asymmetric supercapacitors (ASCs). Asymmetric supercapacitors, namely hybrid supercapacitors, their outstanding advantage is to realize the great working potential window. ASCs are an improvement of the symmetrical supercapacitors, which contain two different type of electrode, a capacitor-type electrode (power source) and a battery-type faradic electrode (energy source), and can enlarge the operating voltage in the whole system by take advantage of the different voltage windows of both electrodes.^8,9^ Therefore, the key to fabricate an ASC is to select suitable negative electrode and positive electrode materials which can work in different potential windows and can be effectively combined together, for the same electrolyte.

Currently, the negative electrodes of ASC choose all kinds of high surface area of carbon materials, among them, activated carbon is the most common used material. Activated carbon material with low-cost, stable performance, good conductivity and easy preparation, is an ideal negative material. Nevertheless, the electrochemical capacitance of an ASC relies mainly on the positive electrode materials. Thus, during past few years, metal-based electrode materials, such as RuO_2_, MnO_2_, Ni_3_S_2_, *etc.*,^10–17^ have been exhibited excellent electrochemical properties and especially high specific capacitance.

However, compared to transition metal oxides, sulfides and selenides which are frequently investigated, up to now, the transition metal tellurides as the electrode material have rarely been reported. In the periodic table, tellurium is in the transition of metal to non-metal, and its physical properties and chemical properties are between metal and non-metal, which means it having good conductivity. And previous studies exhibited most of the metal tellurides possess a superior stability. For instance, a hybrid Te/Au/MnO_2_ was fabricated by Cao *et al.*^18^ and used as supercapacitor electrode with specific capacitance of 930 F g^−1^ and 3% largest loss of the specific capacitance compared with the first cycle during 1000 cycles. Zhou *et al.*^19^ synthesized NiTe rods on Ni-foam by a hydrothermal method with the highest specific capacitance of 804 F g^−1^ and remained 91.3% of initial specific capacitance after 1000 cycles. Some efforts are still needed to enroll conductivity and energy storage ability of tellurium into intrinsic metal telluride based supercapacitors. The cobalt being one of the metal ions with multiple oxidation states (Co^2+^, Co^3+^ and Co^4+^) and high reduction potential, CoTe could serve the purpose of high potential window, high energy density and power density, *etc.* required for supercapacitor. In addition, in analogy to other transition metal chalcogenides, such as Co_3_O_4_,^20,21^ CoS^22^ and Co_0.85_Se,^16^ which has been demonstrated to possess excellent pseudo-capacitive performance, CoTe probably is also a kind of promising electrode material for supercapacitors.

Besides, further to improve the performance of supercapacitors, an innovative approach is explored that introducing redox additive into the electrolytes. Such as VOSO_4_,^23^ KI,^24^ hydroquinone,^25^ indigo carmine^26^ and polysulfide^27^*et al.* The specific capacitance of supercapacitors is notably enhanced as well as energy density due to the additional pseudo-capacitance deriving from redox additives.^28^ Especially, through introducing K_3_Fe(CN)_6_/K_4_Fe(CN)_6_ couple into KOH aqueous solution for NiO electrode at −20 °C, the specific capacitance of NiO rose twice and superior to the performance at room temperature.^29^ Later, the electrochemical property of active carbon-based capacitor has been enhanced through adding two redox additives of K_4_Fe(CN)_6_ and anthraquinone-2,7-disulphonate (AQDS) into the KNO_3_ aqueous solution.^30^ However, this method so far has not been applied to the ASC for further improving its specific capacitance as well as energy density.

Carbon fiber paper (CFP) has been often used as supporter/or current collector owing to its excellent characteristics of good conductive, light-weight, high surface area and chemical inertness.^20,31,32^ In this paper, cobalt telluride (CoTe) nanosheets were successfully synthesized on CFP through a simple *in situ* growth method, and the CoTe electrode displayed favorable electrochemical properties. Then, an asymmetric supercapacitor (ASC) was assembled by employing CoTe as positive electrode and AC (activated carbon) as negative electrode, which could extend voltage window to 1.6 V. Finally, potassium hexacyanoferrate(ii) (K_4_Fe(CN)_6_) served as redox additive was introduced into KOH electrolyte for the ASC to further increase the value of capacitance and energy density.

## 2. Experimental section

### 2.1 Materials

Co(NO_3_)_2_·6H_2_O and Na_2_TeO_3_ were purchased from Aladdin Industrial Corporation. Anhydrous ethanol, N_2_H_4_·H_2_O (80 wt%) and polytetrafluoroethylene (PTFE) aqueous solution (60 wt%) were from Sinopharm Chemical Reagent Co., Ltd. Activated carbon (AC) (surface area 2167 m^2^ g^−1^) was from Fuzhou Yihuan Co., Ltd. Carbon fiber paper was from Cetech Co., Ltd. None of the reagents have been further purified.

### 2.2 Synthesis of CoTe

CoTe electrode material was prepared by hydrothermal process. Typically, the reaction solution of hydrothermal contained 0.3 mmol of Na_2_TeO_3_, 0.3 mmol of Co(NO_3_)_2_·6H_2_O and 55 mL of deionized water. After magnetic stirring, 15 mL of N_2_H_4_·H_2_O was added with vigorous stirring. Then the aqueous solution was transferred to a 100 mL Teflon-lined autoclave. After being stirred evenly, a piece of washed CFP was installed in the autoclave and immersed in reaction solution. The autoclave was sealed and heated at 140 °C for 12 h, and black sample on CFP was obtained from autoclave after hydrothermal process. Finally, product was rinsed several times with water and ethanol, and then dried in vacuum at 60 °C for 12 h. The mass of CoTe on CFP was about 1.2 mg cm^−2^.

### 2.3 Fabrication of asymmetric supercapacitor

The as-prepared CoTe on CPF as the positive electrode and active carbon as the negative electrode were used to construct the asymmetric supercapacitor (ASC) with 3 M KOH aqueous solution as the electrolyte. Activated carbon (80%), PEFT aqueous solution (10%) and acetylene black (10%) was used to synthesis the activated carbon (AC) electrode, which was then pressed on nickel foam and dried at 70 °C for 12 h.

### 2.4 Characterization

Field emission scanning electron microscopy (FESEM) and powder X-ray diffractions (XRD) were used to investigate the morphology and structure characteristics of the samples.

The CPF loaded with CoTe was used as bind-free positive electrode without further treatment. All the electrochemical properties (including CV (cyclic voltammetry), GCD (galvanostatic charge–discharge), EIS (electrochemical impedance spectroscopy)) of CoTe nanosheets electrode were carried out on a CHI660E electrochemical workstation by using 3 M KOH aqueous as electrolyte with three-electrode system. The long cycle life of the CoTe electrode was evaluated using LAND battery test system (CT2001A). The Hg/HgO and platinum plate as reference and counter electrode, and CoTe nanosheets on CFP as working electrode. Furthermore, a two-electrode system was carried out to measurement the electrochemical properties of CoTe//AC ASC in a 3 M KOH aqueous solution. In this process, all the electrochemical measurements of single electrode were performed to value the electrochemical properties of CoTe//AC ASC except EIS test.

The specific capacitance (*C*_m_) of the single electrode expressed by the following eqn (1):^33^1
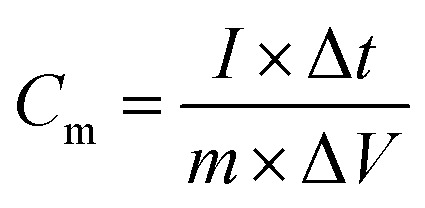
wherein *m* (g) was the mass of the active substance of electrode. Δ*V* (V), *I* (A), and Δ*t* (s) represented the voltage window, the discharge current and the discharge time, respectively.

Other two key factors to evaluation the energy density (*E*, W h kg^−1^) of supercapacitor was calculated using the established eqn (2), and power density (*P*, W kg^−1^) was obtained according to eqn (3):^33^2
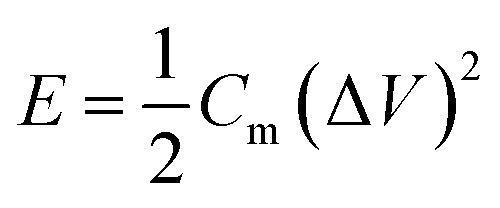
3
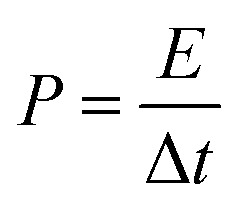


## 3. Results and discussion

### 3.1 Structural and morphological characterization of CoTe

**Fig. 1:**
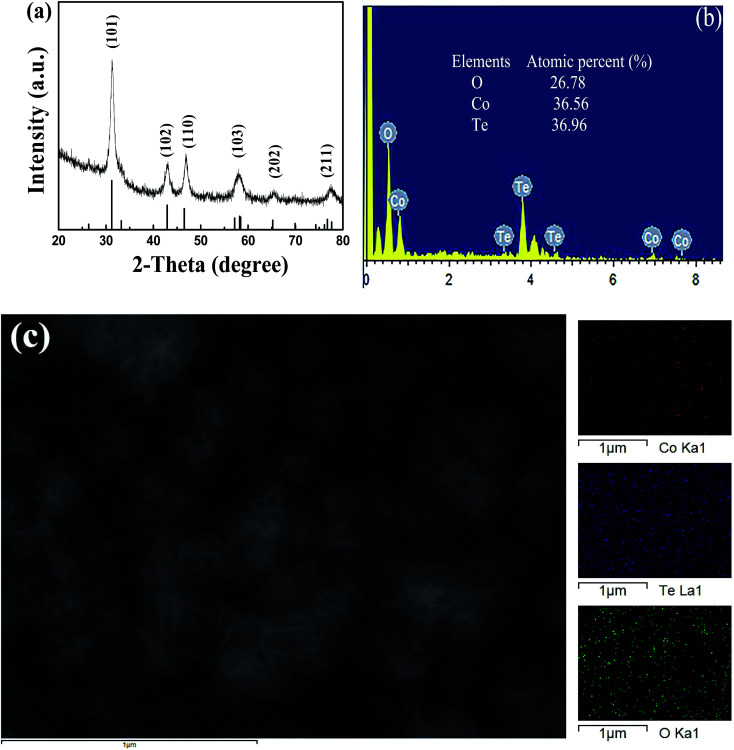
(a) XRD spectrum of CoTe, (b) EDS pattern of CoTe, (c) elemental mappings with different color for different elements.

For the sake of investigate the crystal phase and structure information of products, XRD pattern was measured and shown in Fig. 1(a). The black samples were scratched from the CFP substrate to avoid the effect of carbon signal. Obviously, the six strong diffraction peaks (31.3°, 43.0°, 46.7°, 58.3°, 65.3°, 76.9°) in this pattern can be readily indexed to CoTe (JCPDS no. 34-0420) and no impurity diffraction peak was found. The result of XRD pattern indicated that CoTe had successfully grown on CFP by a facile hydrothermal method. Moreover, energy dispersive spectrometry (EDS) analysis and elemental mapping were also used to study the composition of samples. As shown in Fig. 1(b) and (c), the uniform distribution of cobalt and tellurium were found with their corresponding ratio of 1 : 1 in the sample, indicating the samples mainly composed of CoTe materials. The exist of small amount of oxygen which may come from the absorption of oxygen and moisture on the samples' surface.^34^ The corresponding chemical reactions to form CoTe were as follows^35^

4 2Co^2+^ + 2TeO_3_^2−^ + 3N_2_H_4_·H_2_O → 2Co + 2Te + 3N_2_↑ + 6H_2_O

5 Co + Te → CoTe

**Fig. 2:**
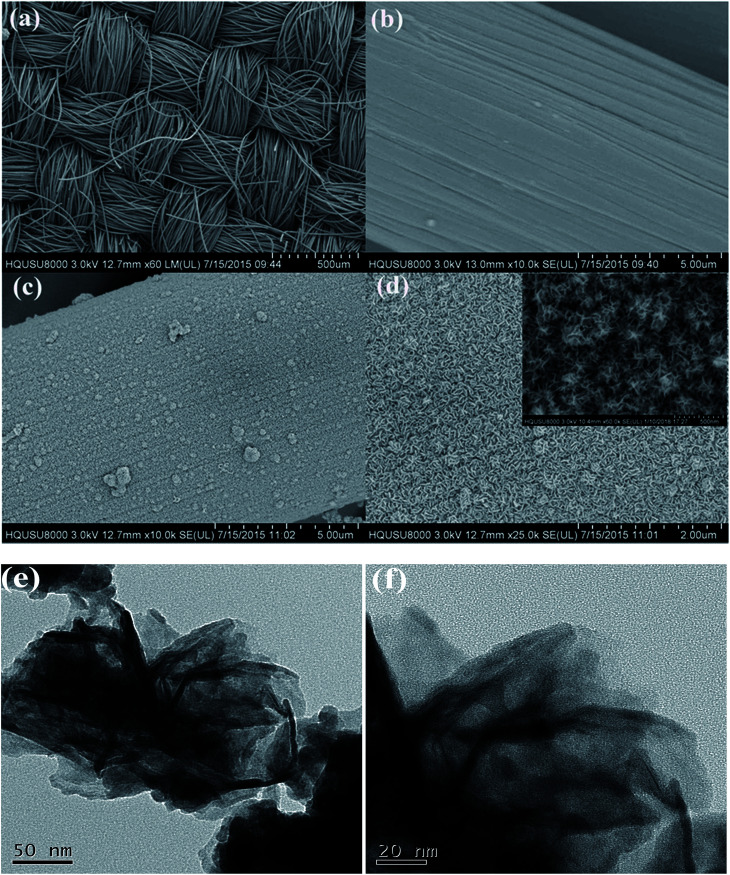
FESEM images of CoTe on CFP and pure CFP: (a) and (b) pure CFP, (c) and (d) CoTe on CFP (the inset was the FESEM image at high magnification); (e) and (f) HRTEM image of CoTe.

The morphology and microstructure of the CFP and CoTe on CFP were observed by FESEM. Fig. 2(a) showed the carbon fiber paper (CFP) was made of countless carbon fibers to weave into an alternate network structure, which could provide good mechanical flexibility and fine transmission performance. That is to say, electron still could successfully pass the network structure in the case of a minority of carbon fiber fracture. Before participating in the hydrothermal reaction, the surface of CFP was smooth as shown in Fig. 2(b), which was carefully washed by alcohol, acetone and distilled water in turn. Fig. 2(c) and (d) showed the FESEM images of as-synthesized CoTe on the CFP substrate at low and high magnification, respectively. The surface of the CFP substrate became rough after the hydrothermal reaction which could be clearly seen in Fig. 2(c). In order to observe the microstructure of CoTe, we employed the FESEM image of high magnification (Fig. 2(d)). As can be seen, the sheet-like CoTe arrays uniformly covered the surface of CFP and interconnected with each other, which could conducive to the full contact of the electrolyte with the active material surface and promote the rapid, reversible redox reaction of the electrode, thereby obtaining a larger pseudocapacitance. Transmission electron microscopy (TEM) measurements were carried out to further investigate the structure of the as-synthesized CoTe nanosheets. As can be seen from Fig. 2(e) and (f), the CoTe nanosheets exhibit interconnected morphology with transparent feature, indicating the ultrathin nature.

**Fig. 3:**
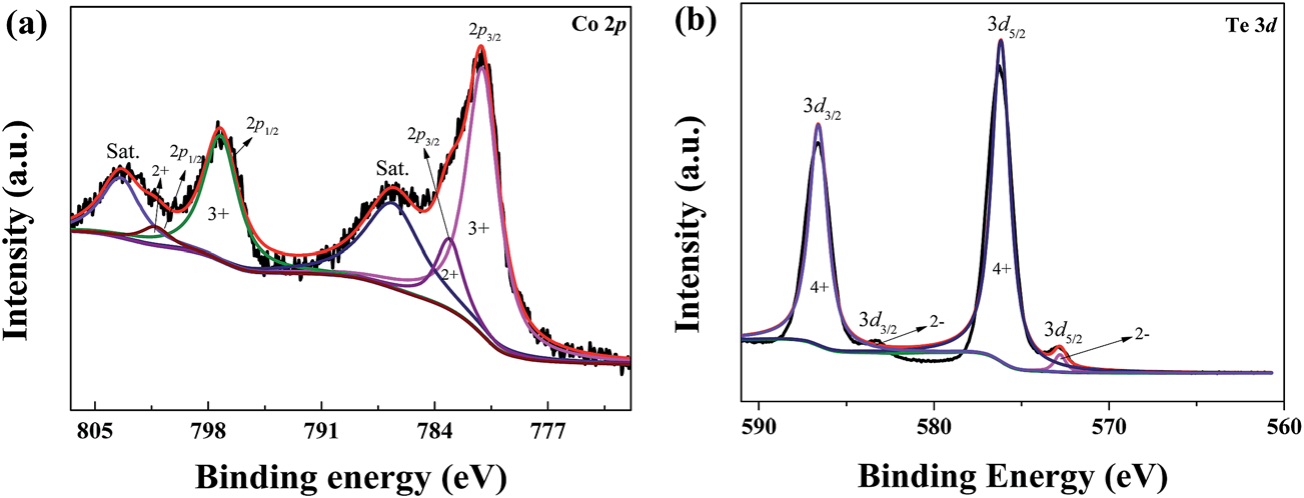
XPS spectra of the (a) Co 2p region, (b) Te 3d region.

To further conform that the CoTe was successfully prepared using this hydrothermal method, X-ray photoelectron spectroscopy (XPS) was performed to analyze the surface elements and their valence state of the CoTe, and the obtained results were shown in Fig. 3. For Co 2p spectrum (as shown in Fig. 3(a)), the binding energy at 783.08 eV, 781.05 eV, 801.26 eV, and 797.23 eV were Co^2+^ 2p_3/2_, Co^3+^ 2p_3/2_, Co^2+^ 2p_1/2_ and Co^3+^ 2p_1/2_, respectively, suggesting that the Co atom in the as-synthesized CoTe nanosheets had two valence states (tetrahedral Co^2+^ and octahedral Co^3+^ contributing to 2p spectral profile).^36^ For the Te 3d region (Fig. 3(b)), the peaks at 572.8 eV in Te 3d_5/2_ and 583.3 eV in Te 3d_3/2_ were related to the characteristics of Te^2−^, and the peaks at 576.2 eV in Te 3d_5/2_ and 586.6 eV in Te 3d_3/2_ corresponds to the characteristics of Te^4+^.^37^ The results of XPS spectrum illustrated the existence of CoTe nanosheets.

### 3.2 Electrochemical performance of CoTe electrode

**Fig. 4:**
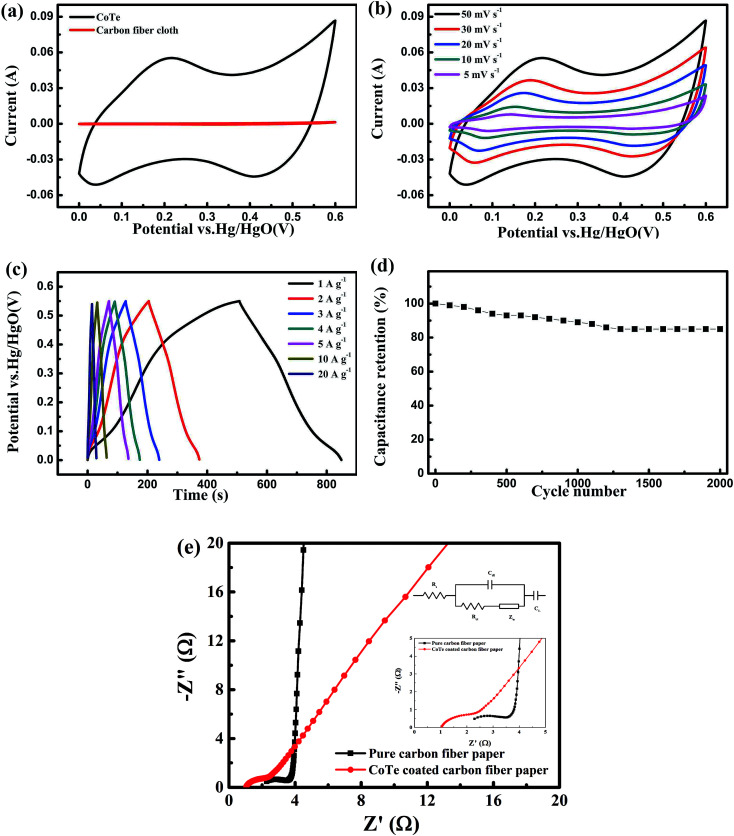
Electrochemical performances of the CoTe electrode: (a) CV curves of the CFP substrate and CoTe electrode at a scan rate of 100 mV s^−1^; (b) CV curves of the CoTe electrode at different scan rates; (c) GCD curves at different current densities; (d) cycling performances during 2000 cycles at current density of 1 A g^−1^; (e) corresponding Nyquist plots for composite electrodes prepared by different doping ratios of Se doped NiTe (the inset were the enlarged curves at a high frequency range and equivalent circuit diagram).

The pseudocapacitive properties of as-prepared carbon fiber paper supported CoTe nanosheets electrode were investigated using CV, GCD and long-term cycling stability tests. In order to study the contribution of the CFP substrate for the total electrode, the CV curves of CFP and CoTe electrode in 3 M KOH solution at a voltage window of 0 to 0.6 V with scan rate of 100 mV s^−1^ by a three-electrode system were exhibited in Fig. 4(a). Obviously, the CV curve of pure CFP electrode was almost a straight line, indicating that the capacitance behavior from CFP could be neglected, the electrochemical capacitance of as-synthesized electrode was dominated by CoTe nanosheets.^38^Fig. 4(b) showed CV curves of the CoTe electrode at different scan rates, where two pairs of redox peaks appeared in each curve, which correspond to the typical faradic reaction among transformation of Co^2+^/Co^3+^/Co^4+^ in alkaline solution, revealing that CoTe electrode had the pseudo-capacitance performance and was a Faraday-type supercapacitor electrode material instead of the electric double layer electrode material.^24,39^ According to Fig. 4(a), the two pairs of redox peaks at about 0.21 V and 0.03 V, 0.50 V and 0.41 V, which suggests that the capacitive characteristics are mainly manifested as pseudo-capacitance characteristics. The two peaks could be due to the following redox reactions in analogy with CoS^39^ and Co_0.85_Se.^20^

6 CoTe + OH^−^ ↔ CoTeOH + e^−^

7 CoTeOH + OH^−^ ↔ CoTeO + H_2_O + e^−^

Fig. 4(c) presented the GCD curves of CoTe electrode at different current densities. All of the curves were to keep good symmetry during the charge–discharge process, which indicated CoTe electrode had a good electrochemical capacitive characteristic and highly reversible nature.^16,40^ A sequence of specific capacitances (*C*_m_) were evaluated based on eqn (1), the *C*_m_ of CoTe electrode was increasing from 553.4 F g^−1^ to 622.8 F g^−1^ with the current density ranging from 20 A g^−1^ to 1 A g^−1^. Fortunately, with the current density increase to 20 A g^−1^, still 88.9% of the *C*_m_ remained relative to the value of 1 A g^−1^, indicating that the CoTe nanosheets supported on CFP electrode had a high rate performance.^38,41^ The cyclic stability of CoTe electrode was evaluated by a continuous charge–discharge process at 1 A g^−1^ for 2000 cycles. It can be seen from Fig. 4(d), at the first 1000 cycles, the capacitance of CoTe electrode decreased gradually ∼10%, and then remained stable value after 1300 cycles. And after 2000 cycles, the capacitance of the CoTe electrode had still 85% compared with pristine value, indicating that its excellent cycle stability. Moreover, electrochemical impedance spectroscopy (EIS) measurements could be utilized to measure ion transfer properties and electrical conductivity of the different electrode materials. Fig. 4(e) presented the Nyquist impedance plots tested in standard three-electrode system in the frequency range from 10^−2^ to 10^5^ Hz, and the inset was the corresponding equivalent circuit diagram. The intercept at the part (*Z*′) is the internal resistance (*R*_s_), which contains the intrinsic resistance of electrode materials, the contact resistance at the electrode/electrolyte interface and the bulk resistance of electrolyte, and the semicircle at high frequencies region relates to the faradaic charge-transfer resistance (*R*_ct_). Besides, the straight line in the low frequency region represents the Warburg impedance (*Z*_w_), indicating that the charge–discharge process of electrode is controlled by ion diffusion.^5,42,43^ Additionally, *C*_L_ and *C*_dl_ are the limit capacitance and the double-layer capacitance.^28^ The fitted *R*_s_, *R*_ct_ and *Z*_w_ of pure carbon fiber paper (CFP) and CoTe coated carbon fiber paper were listed in Table 1.

**Table 1:** The fitted impedance of pure CFP and CoTe coated CFP

Sample	*R*_s_ (Ω cm^2^)	*R*_ct_ (Ω cm^2^)	*W*, *Y*_o_ (S s^1/2^ cm^−2^)
Pure CFP	2.29	3.89	2.14 × 10^−4^
CoTe coated CFP	1.04	1.86	1.25 × 10^−3^

From above results, it can be seen that the value of *R*_s_ and *R*_ct_ of CoTe coated CFP was much small than those value of pure CFP, which means the conductive of CoTe coated CFP was superior than pure CFP. While the *Z*_w_ of CoTe coated CFP was larger than pure CFP, which lead to a better semi-infinite diffusion of the ions in the electrode.^36^

The outstanding electrochemical performance of CoTe nanosheet electrode was attributed to the following reasons. Firstly, the orderly CoTe nanosheets structure gives rise to a high area, not only improving the utilization of active materials but also offering a large number of electrically active sites which were conducive to adsorb ions.^44^ Besides, the open space between the nanosheets could promote the electrolyte penetration into the inner of the electrode.^42,44^ Lastly, the “dead surface” of the traditional slurry-derived electrode could be avoid through directly grown CoTe nanosheets on CFP.^5,44^

### 3.3 Asymmetric supercapacitor

**Fig. 5:**
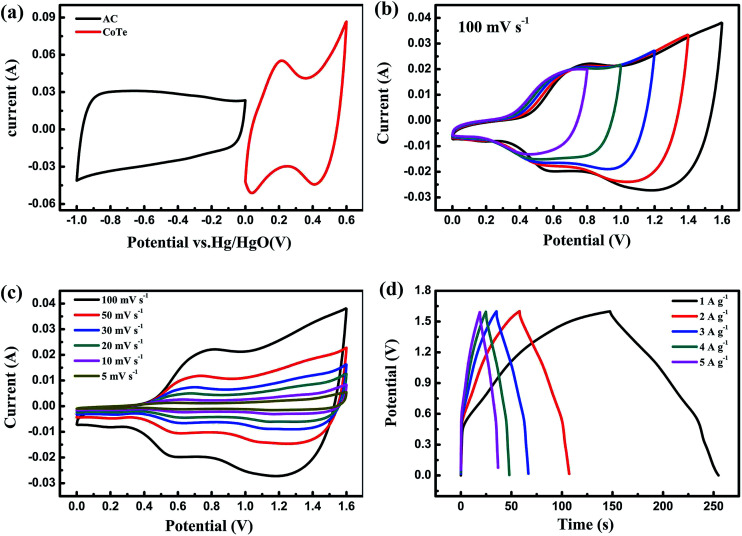
(a) CV curves of the CoTe and AC electrodes collected at 100 mV s^−1^ in a three-electrode system; (b) CV curves of CoTe//AC ASC at different potential windows at 100 mV s^−1^; (c) CV curves of CoTe//AC ASC at different scan rates; (d) GCD curves of CoTe//AC ASC at different current densities.

In order to further research the potential application of CoTe nanosheets on CFP electrode for energy storage, AC as negative electrode and CoTe as the positive electrode were used to fabricate an asymmetric supercapacitor (ASC) with 3 M KOH as electrolyte. The complementary CV curves of AC and CoTe electrode, including AC electrode and CoTe electrode occupy different voltage windows were −1 to 0 V and 0 to 0.6 V, respectively, were shown in Fig. 5(a). Thus, an expanded voltage window of 1.6 V can be achieved by assembled CoTe and AC electrode. To maximize the performance of ASC, the mass loading of AC was determined by balancing the charges stored in each electrode. The mass specific capacitance of CoTe and AC electrode was calculated to be 622.8 F g^−1^ and 228.4 F g^−1^, respectively, at the current density of 1 A g^−1^. Therefore, the mass loading of AC in CoTe was calculated based on eqn (8).^5,45^8
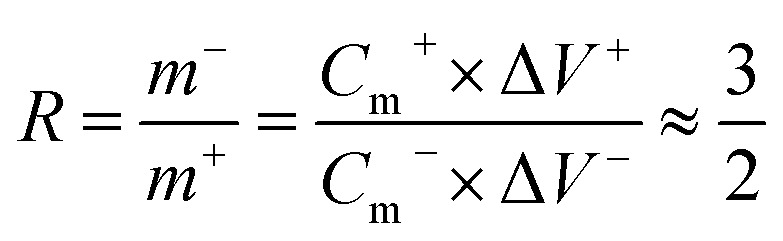
where *m*^−^ and *m*^+^ were the mass of activated materials, *C*_m_^−^ and *C*_m_^+^ represent mass specific capacitance, Δ*V*^−^ and Δ*V*^+^ were the potential windows of negative and positive electrodes, respectively.

With a scan rate of 100 mV s^−1^, the CV curves of the CoTe//AC ASC at various voltage windows from 0.8 V to 1.6 V were displayed in Fig. 5(b). It is noted that all curves remain similarity and stable with increasing of the voltage window, indicating the CoTe//AC ASC can be stably operated at a high voltage of 1.6 V as expected.^45^Fig. 5(c) showed the CV curves of CoTe//AC ASC at different scan rates. Due to the pseudo-capacitive nature of the CoTe nanosheet electrode, strong redox peaks could be clearly observed at each curve. At various current densities, the GCD curves of CoTe//AC ASC in a voltage window from 0 to 1.6 V were displayed in Fig. 5(d). The GCD curves were nearly symmetrical, indicating the excellent reversibility of CoTe//AC ASC.^45^

**Fig. 6:**
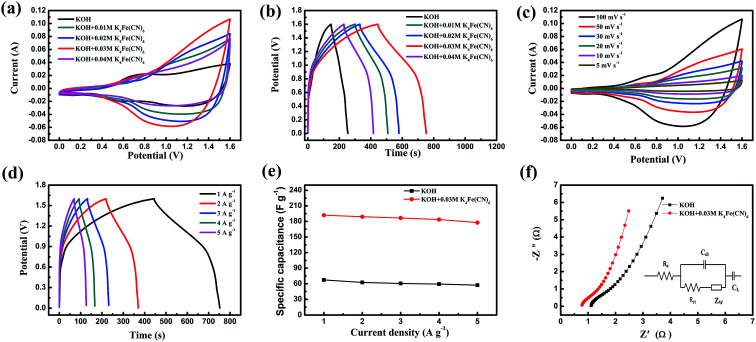
(a) CV curves of ASCs at 100 mV s^−1^ in KOH and K_4_Fe(CN)_6_ + KOH; (b) GCD curves of ASCs at 1 A g^−1^ in KOH and K_4_Fe(CN)_6_ + KOH; (c) CV curves of ASC at different scan rates in K_4_Fe(CN)_6_ + KOH; (d) GCD curves of ASC at different current densities in K_4_Fe(CN)_6_ + KOH; (e) the mass specific capacitance of ASCs at different current densities in KOH and K_4_Fe(CN)_6_ + KOH; (f) Nyquist plots of ASCs in KOH and K_4_Fe(CN)_6_ + KOH.

In order to further improve the performance of CoTe//AC ASC, K_4_Fe(CN)_6_ as a single redox additive was introduced into alkaline electrolyte (3 M KOH). To obtain the optimized electrochemical performance of the CoTe//AC ASC, the effects of different concentration of K_4_Fe(CN)_6_ adding into the KOH electrolyte were investigated. Fig. 6(a) exhibited CV and GCD curves of CoTe//AC ASC with different concentration of K_4_Fe(CN)_6_ adding into the KOH electrolyte at a scan rate of 100 mV s^−1^ with a potential window of 0–0.55 V. The area of the CV curves represents their capacitances. It is noticed that the area of CoTe//AC ASC with 0.03 M K_4_Fe(CN)_6_ + 3 M KOH was far larger than those of others, which means larger specific capacitance of CoTe//AC ASC with 0.03 M K_4_Fe(CN)_6_ + 3 M KOH. This fact could also be verified by GCD test. Fig. 6(b) exhibited GCD curves of different electrode samples at current density of 1 A g^−1^. Obviously, the discharge time of CoTe//AC ASC with 0.03 M K_4_Fe(CN)_6_ + 3 M KOH was longest at the same current density. As a result, the K_4_Fe(CN)_6_ with 0.03 M was the optimal concentration. In addition, the electrolyte containing 0.03 M K_4_Fe(CN)_6_ exhibited additional redox peak which was due to the possible redox reaction between the redox pair of Fe(CN)_6_^3−^/Fe(CN)_6_^4−^. The possible redox reaction in ASC was given below,^46^

9 Fe(CN)_6_^4−^ ↔ Fe(CN)_6_^3−^ + e^−^

Moreover, by adding K_4_Fe(CN)_6_ into pristine electrolyte, the CV curve with the enclosed area also increased, which implied that the capacitance was enhanced.^28^Fig. 6(c) was the CV loops of the CoTe//AC ASC at different scan rates in the K_4_Fe(CN)_6_ + KOH solution, which could easily find that almost all the CV curves keeps the same with increasing the scan rate. Galvanostatic charge–discharge test was also executed to measure the specific capacitance value of ASC in K_4_Fe(CN)_6_ + KOH electrolyte, as presented in Fig. 6(d). The corresponding calculated value of specific capacitances in K_4_Fe(CN)_6_ + KOH electrolyte, as shown in Fig. 6(e), was increased from 177.8 F g^−1^ to 192.1 F g^−1^ at current densities changed from 5 A g^−1^ to 1 A g^−1^, respectively. The calculated results displayed that the specific capacitance of CoTe//AC ASC with adding extra redox additive K_4_Fe(CN)_6_ into KOH was huge increased and almost triple higher than the specific capacitance of ASC in pristine electrolyte (57.3 F g^−1^ to 67.3 F g^−1^ with current density from 5 A g^−1^ to 1 A g^−1^). Therefore, the performance of ASC was enhanced by introducing 0.03 M K_4_Fe(CN)_6_ into electrolyte through its possible redox reaction (eqn (9)). EIS was performed to further explore the improved capacitive behavior of the CoTe//AC ASC in K_4_Fe(CN)_6_ + KOH solution. The resulting Nyquist plots were presented in Fig. 6(f), and the inset was the fitted equivalent circuit. As is shown in Fig. 6(f), the CoTe//AC ASC in K_4_Fe(CN)_6_ + KOH solution had smaller intercept of the real axis and semicircle compared with CoTe//AC ASC in KOH solution, which was verified by the fitted value of *R*_s_ and *R*_ct_, meaning the small bulk resistance and lower electronic resistance of this improved electrolyte. According to the fitted results, the ASC in K_4_Fe(CN)_6_ + KOH electrolyte not only had the lower internal resistance (*R*_s_, 0.77 Ω *vs.* 1.11 Ω), but also possess of a smaller interfacial charge transfer resistance (*R*_ct_, 0.49 Ω *vs.* 0.56 Ω), which means adding K_4_Fe(CN)_6_ into KOH electrolyte could lead to an increase of the conductivity which has been confirmed by EIS test. This was the positive effect which was the reason why the electrochemical performance of ASC had significantly promoted by introducing K_4_Fe(CN)_6_ as redox additive into pristine electrolyte.

**Fig. 7:**
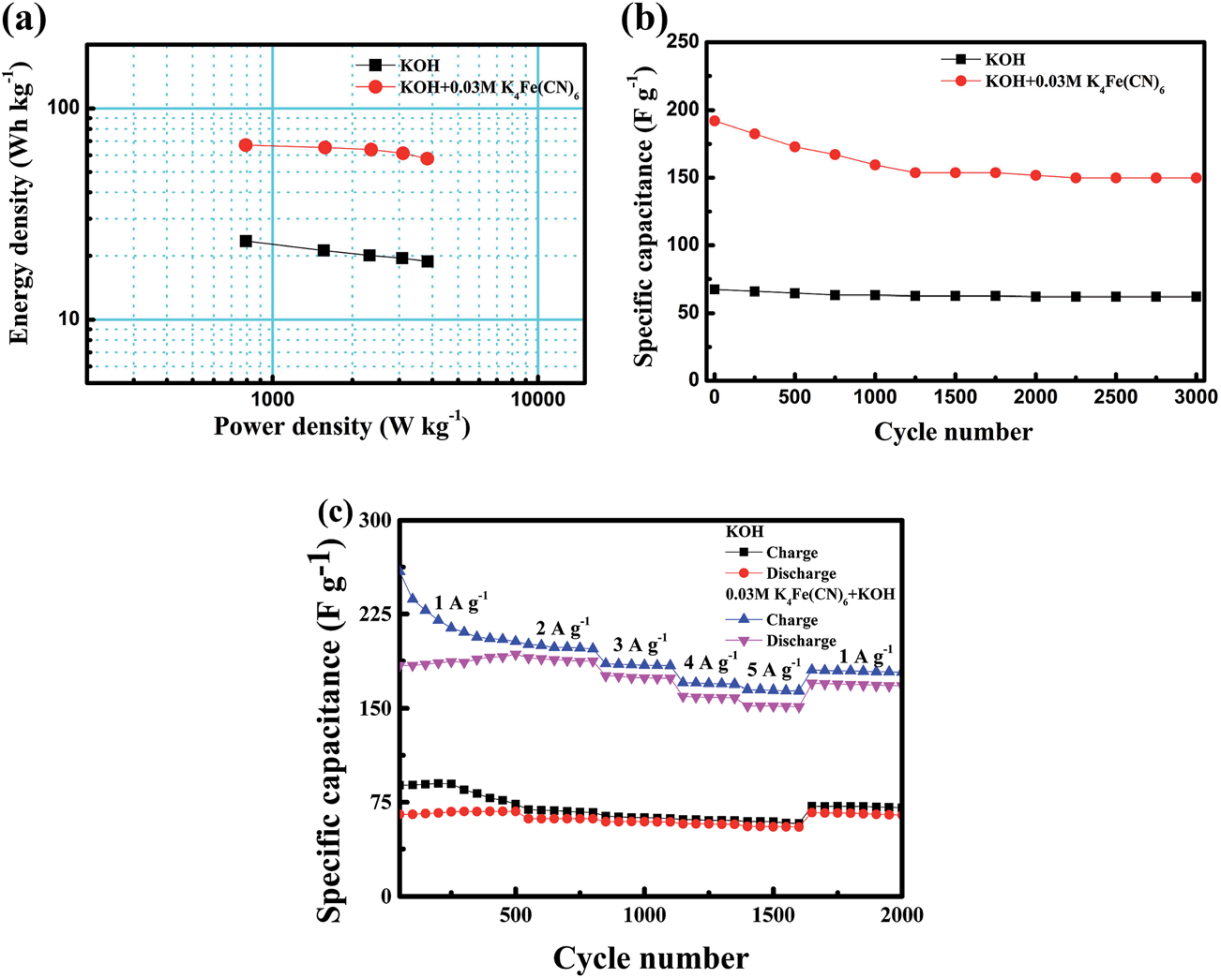
(a) Ragone plots of ASCs in KOH and K_4_Fe(CN)_6_ + KOH electrolyte; (b) cycling performance of ASCs in KOH and K_4_Fe(CN)_6_ + KOH electrolyte at current density of 1 A g^−1^; (c) cycling performance at various rates from 1 to 5 A g^−1^ after 50 cycling at 1 A g^−1^.

The energy and power densities of the CoTe//AC asymmetric supercapacitors were calculated according to the GCD curves and their Ragone plots were shown in Fig. 7(a). The energy densities of CoTe//AC ASC with redox additive of K_4_Fe(CN)_6_ into KOH were clearly higher than those of ASC without redox additive. And the maximum energy density of ASC in K_4_Fe(CN)_6_ + KOH electrolyte could reach 67.0 W h kg^−1^, which was nearly threefold increase than pristine electrolyte (23.5 W h kg^−1^) at almost the same power density (∼793.5 W kg^−1^). The highest energy density was superior to those earlier reported for Te-based or Co-based components and (as is shown in Table 2), such as NiTe//AC ASC in KOH electrolyte (33.6 W h kg^−1^),^19^ 1T′-MoTe_2_//AC in KOH electrolyte (56.4 W h kg^−1^),^37^ Co_0.85_Se//AC in KOH (45.0 W h kg^−1^)^16^ and Co_0.85_Se//AC in KOH (39.7 W h kg^−1^).^47^

**Table 2:** The comparison energy density of Te-based components

Components	Electrolyte	Energy density (W h kg^−1^)
CoTe	3 M KOH	23.5
CoTe	0.03 M K_4_Fe(CN)_6_ + 3 M KOH	67.0
NiTe	3 KOH	33.6
1T′-MoTe_2_	2 M KOH	56.4
Co_0.85_Se	3 KOH	45.0
Co_0.85_Se	3 KOH	39.7

Furthermore, the long cycle stability was test to evaluated the applicability of CoTe//AC ASCs, which was shown in Fig. 7(b). It can generally find that the ASC without redox additive exhibited excellent cycling performance with 92.3% retention of capacitance after 3000 cycles, which owing to the prominent electrochemical stability of both AC and CoTe electrodes. The specific capacitance of ASC in K_4_Fe(CN)_6_ + KOH electrolyte had a significantly reduced in the first 1250 cycles, but still remains 80.7% of initial capacitance after 3000 cycles. Meanwhile, the capacitance value of ASC with redox additive reaches 149.8 F g^−1^ and significantly higher than that of the ASC without K_4_Fe(CN)_6_, indicating that the ASC with K_4_Fe(CN)_6_ had a great prospect of the application. The capacitance retention rate of ASC containing redox additive was distinctly lower than those without K_4_Fe(CN)_6_, which could be attributed to the reduction of the activity of the redox additive in the redox process.^25,26^ This could be verified by rate capability of ASCs with different electrolyte at different current densities. Fig. 7(c) revealed the current density dependence of the cycling performance. During the process, five steps of charge–discharge rates were changed successively from 1 to 5 A g^−1^. At the first 500 cycles with charge–discharge current densities of 1 A g^−1^, both ASCs showed unsteady specific capacitances with a relatively low coulombic efficiency. In the following 1100 cycles at large current densities, the ASC with KOH as electrolyte demonstrates a stable performance at each situation while the ASC with K_4_Fe(CN)_6_ + KOH as electrolyte shows a little decay. When the current turns back to 1 A g^−1^, a fully recovered ASC with KOH as electrolyte with a coulombic efficiency of ≈91.6% was observed in the following 400 cycles while the retention capacitance of ASC with K_4_Fe(CN)_6_ + KOH as electrolyte is calculated to be ≈88.2% with a coulombic efficiency of 94.9%.

## 4. Conclusion

In summary, CoTe nanosheets were successfully fabricated on conductive carbon fiber paper by one step hydrothermal process and directly served as supercapacitor electrode. At current density 1 A g^−1^, CoTe electrode exhibited highest *C*_m_ of 622.8 F g^−1^ and excellent long-term cycle stability. In addition, an asymmetric supercapacitor based on CoTe as positive electrode and AC as negative electrode, respectively, had been assembled. The as-fabricated ASC could be operated stably in KOH, and K_4_Fe(CN)_6_ + KOH electrolytes with a high voltage of 1.6 V. The redox additive could provided additional pseudo-capacitance by redox reaction. ASC with K_4_Fe(CN)_6_ + KOH delivered the specific capacitance of 192.1 F g^−1^ with energy density of 67.0 W h kg^−1^ at 1 A g^−1^ was almost threefold increase than ASC with pristine electrolyte (67.3 F g^−1^, 23.5 W h kg^−1^). Therefore, it was a highly promising pathway by introducing the redox additive to improve the performance of ASC for practical application.

## Conflicts of interest

There are no conflicts to declare.

## Supplementary Material
